# Opsoclonus-myoclonus syndrome with severe clinical course and beneficial outcome: A case report

**DOI:** 10.1097/MD.0000000000025261

**Published:** 2021-04-09

**Authors:** Ewa Koziorowska-Gawron, Magdalena Koszewicz, Joanna Bladowska, Maria Ejma, Slawomir Budrewicz

**Affiliations:** a Department of Neurology; b Department of General, Interventional Radiology and Neuroradiology, Wroclaw Medical University, Wroclaw, Poland.

**Keywords:** ataxia, case report, myoclonus, opsoclonus, pregnancy

## Abstract

**Rationale:**

Opsoclonus-myoclonus syndrome (OMS) is a rare immune-mediated movement disorder, mostly of paraneoplastic or idiopathic origin. The disease usually has an acute onset, serious course and leads rapidly to disability in adult patients. To the best of our knowledge, this is the fourth presented case of OMS with a severe course and complete reversibility of neurological symptoms in a pregnant woman. This report includes videos and a literature review.

**Patient concerns:**

A 30-year-old woman in the 12th week of pregnancy developed severe nausea and vomiting, after several days balance and gait disorders appeared. On admission to hospital, neurological examination revealed opsoclonus, dysarthria, myoclonic jerks with ataxia of the trunk and limbs with inability to sit, stand or walk.

**Diagnosis:**

Well-known causes of OMS were excluded. Although in our patient the idiopathic origin of the disorder was taken under consideration, diagnosis of opsoclonus-myoclonus related to the pregnancy was highly likely.

**Interventions:**

After administration of steroids and benzodiazepines the patient improved.

**Outcomes:**

In the 6th month of pregnancy, after termination of immunotherapy, she recovered completely and was able to sit, stand and walk independently. In the 39th week of pregnancy, she delivered a healthy child.

**Lessons:**

We confirm that understanding of clinical symptoms and rare causes of OMS contributes to early diagnosis and therapy, which ensures an optimal outcome. One probable cause of OMS could be a physiological change to immune system regulation during pregnancy. The relationship between OMS and pregnancy remains uncertain and needs further investigation.

## Abstract 2

Supplemental Digital Content is available in the text

## 1 Introduction

In adults, opsoclonus-myoclonus syndrome (OMS) is a rare, mostly immune-mediated movement disorder, rapidly leading to disability. The onset of symptoms is acute or subacute, with spontaneous, saccadic eye movements (opsoclonus), myoclonic jerks affecting different parts of the body, cerebellar ataxia and occasionally behavioral and mood changes, as well as cognitive disturbances.^[1,2,3,4,5,6]^ Supposed diagnostic criteria for OMS require fulfilment of 3 of 4 features: opsoclonus, myoclonus or ataxia, behavioural changes or sleep disturbances, tumorous conditions, or presence of antineuronal antibodies.^[7]^ Delayed diagnosis of OMS has a negative impact on the prognosis and final outcome.^[8]^

The term opsoclonus was first used in 1913 by Polish neurologist Kazimierz Orzechowski, who later in 1927 identified the association between opsoclonus and myoclonus.^[9]^ Opsoclonus is defined as involuntary, multidirectional, conjugate arhythmic eye movements with horizontal, vertical, and torsional components, high frequency (10–15 Hz) and large amplitude. Opsoclonus appears during fixation, slow smooth and convergence eye movements, as well as eyelid closure.^[1]^ Myoclonus, described as sudden arrhythmic jerks, may involve head, neck, trunk and limbs and can be present in certain positions (posture-induced myoclonus), or during action (movement-induced myoclonus). Different kinds of stimuli, including emotional reactions, exacerbate myoclonus. Neither opsoclonus nor myoclonus disappear during sleep.^[1,3]^ Cerebellar symptoms present in OMS include dysarthria and ataxia, which usually predominates in trunk and lower limbs. Both myoclonus and ataxia are responsible for sitting, standing and walking disability.^[1,3]^ Behavioral changes, such as inattention, disorientation, affective dysregulation or anxiety, and cognitive disturbances seem to have an encephalopathic background.^[1]^

To the best of our knowledge, we are here presenting the fourth case of OMS with a severe course and good final outcome after early therapeutic intervention in a pregnant woman. We include videos and a literature review.

## 2 Case report

A 30-year-old woman in the 12th week of pregnancy, with no medical history except a history of 3 miscarriages and 1 healthy child delivery, developed severe nausea and vomiting. Several days later dizziness, balance and gait disorders appeared and these were accompanied by rapid, irregular eye movements causing fixation problems. On admission to hospital, neurological examination of the patient revealed opsoclonus, dysarthria, myoclonic jerks within the neck and limbs, ataxia of the trunk and limbs with inability to sit, stand or walk. There was no relevant information in the family's medical history. Additional video files show more details of the clinical course [see supplemental video files 1 (opsoclonus and myoclonus. A 30-year-old woman in the 3rd month of pregnancy with multidirectional, arhythmic eye movements during fixation and looking in horizontal and vertical planes (opsoclonus) with myoclonic jerks (myoclonus) within the neck.], http://links.lww.com/MD/F962, 3 (Truncal ataxia. A 30-year-old woman in the 3rd month of pregnancy with truncal ataxia causing inability to sit.), http://links.lww.com/MD/F964, 5 (Truncal and lower limb ataxia with myoclonus. A 30-year-old woman in the 3rd month of pregnancy with standing and walking disability due to truncal ataxia and lower limb ataxia with myoclonus.), http://links.lww.com/MD/F966). Muscle strength, tendon reflexes, and sensation were normal; both meningeal and pyramidal signs were absent.

Brain magnetic resonance (MR) was performed using a 1.5T MR scanner in a short protocol (dedicated to pregnant patients) including T1- and T2-weighted, FLAIR and diffusion weighted images. No pathologies were found in brain MR imaging. In electro-encephalography, non-specific generalized slowdown of background activity with bilateral paroxysmal theta waves was registered. The amplitude and latency of visual evoked potentials were within normal limits. Considering pregnancy as a relative contra-indication for computer tomography, we performed ultrasonography, which showed no changes in the breast, abdomen and pelvis. Obstetric examination, including ultrasonography, revealed no abnormalities and confirmed physiological foetus development. Cerebro-spinal fluid (CSF) examination showed mild lymphocytic pleocytosis (7 cell/mm3) and a high IgG index with CSF-exclusive oligoclonal IgG bands. Routine laboratory serum investigations (including blood count, chemistry, urinalysis, thyroid hormones, and antibodies) were normal. Neither serum onconeuronal (anti-Hu, anti-Yo, anti-Ri, anti-CV2.1, anti-Ma/Ta, anti-Tr, anti-amphiphysin) nor antineuronal (anti- glutamic acid decarboxylase, anti- myelin-associated glycoprotein, anti- N-methyl-D-aspartate receptors, anti-GlyR) antibodies were found. Tumour markers (carcino-embryonic antigen, carcinoma antigen 19–9, carcinoma antigen 15–3, carcinoma antigen 125, alpha-fetoprotein) and autoantibody screen (anti-nuclear antibodies, rheumatoid factor, complement component 3, complement component 4) were within normal limits. Neither antiviral [HIV (human immunodeficiency virus), cytomegalovirus, herpes simplex virus, varicella zoster virus, EBV, hepatitis C virus, hepatitis B virus] nor bacterial (Borrelia burgdorferi, Mycoplasma pneumoniae) antibodies were detected. Antiphospholipid syndrome (lupus anticoagulant, anticardiolipin antibodies), deficiency in natural anticoagulants (protein C, S, antithrombin III), and defects in copper and ceruloplasmin metabolism were excluded.

We excluded well-known causes of OMS. Although in our patient the idiopathic origin of the disorder was taken under consideration, diagnosis of opsoclonus-myoclonus related to the pregnancy was highly likely.

The patient was treated with oral methylprednisolone, 40 mg per day for 2 weeks, and simultaneously, as symptomatic treatment, with clonazepam 1 mg per day for a week with good tolerability and no adverse events. After a week, we observed a gradual improvement; the patient felt much better, she stopped vomiting, and opsoclonus, myoclonus and ataxia definitely diminished. An improvement was noticed in her ability to sit, stand and walk. We gradually discontinued methylprednisolone within 3 months without any additional complications. After termination of immunotherapy, in the 6th month of pregnancy, the patient recovered completely and was able to sit, stand and walk independently. Additional video files show this more detail (see supplemental video files 2 (Correct fixation and normal eye movements. The same 30-year-old woman in the 6th month of pregnancy with correct fixation and normal eye movements.), http://links.lww.com/MD/F963, 4 (Accurate coordination of trunk. The same 30-year-old woman in the 6th month of pregnancy with accurate coordination test of trunk.), http://links.lww.com/MD/F965, 6 (Normal standing and walking. The same 30-year-old woman in the 6th month of pregnancy with normal standing and walking without assistance.), http://links.lww.com/MD/F967). In the 39th week of pregnancy, a healthy child was born by caesarean section and the patient remained asymptomatic at the 6-month follow-up.

## 3 Discussion

### 3.1 Pathophysiology of opsoclonus

The pathophysiological mechanisms responsible for generating opsoclonus remain unclear and there are several theories explaining the phenomenon. There is a probable association between omnipause neurones (OPNs) in the pontine nucleus raphe interpositus in the brainstem and opsoclonus. In physiological conditions, OPNs are responsible for inhibition burst neurons in the paramedian pontine reticular formation and rostral interstitial nucleus of Cajal. The inhibition of burst neurons prevents generation of saccades.^[10]^ Opsoclonus might be a consequence of impairment of OPNs, but this has not been proved in either physiological or histopathological studies.^[11]^ According to the brainstem theory, opsoclonus generation is due to membrane dysfunction of saccadic burst neurons. The membrane alterations of burst neurons cause extreme susceptibility to excitation after OPN inhibition or inefficient OPN inhibition.^[12]^ Based on the cerebellar hypothesis, Purkinje cells in the dorsal vermis have inhibitory projections to the fastigial nucleus in the cerebellum. Incorrect inhibition (disinhibition) of fastigial nucleus could lead to opsoclonus, as evidenced in histopathological studies and functional magnetic resonance, single photon emission-computed tomography, and 18-fluoro-2-deoxyglucose-positron emission tomography in patients with OMS.^[10,11,13–15]^

### 3.2 Immunological features of opsoclonus-myoclonus syndrome

Adult-onset OMS is mostly an immune-mediated disease of paraneoplastic or idiopathic origin, triggered by molecular mimicry or unknown causes. OMS could develop in the course of metabolic, toxic, or structural impairment of the central nervous system.^[1–3,6,16,17]^ In both paraneoplastic opsoclonus-myoclonus syndrome (P-OMS) and idiopathic opsoclonus-myoclonus syndrome (I-OMS) humoral and cell-mediated immune mechanisms are involved, with the presumed dominant role of antibodies.^[5,6,10]^ In some patients with P-OMS, a variety of onconeuronal antibodies are found (anti-Hu, anti-Yo, anti-Ri, anti-CRMP-5/CV2, anti-Ma1, anti-Ma2, anti-Tr, anti-Zic-4, anti-amphiphysin), although the majority of patients are seronegative (1, 3, 4, 5, 6, 18). Achieved remissions and a lack of changes in neuropathological examination in immune-mediated OMS suggest transient neuronal dysfunction due to autoantibodies binding antigens residing in the synapse or on the cell surface.^[10,18]^ Antibodies against known neuronal surface antigens such as glycine receptors (GlyR), gamma-aminobutiric acid type B receptors, N-methyl-D-aspartate receptors and di-peptidyl-peptidase-like protein-6 are identified in serum and CSF of patients with P-OMS and I-OMS. However, even relatively frequent antibodies to GlyR are not sufficiently sensitive or specific to be biomarkers of OMS. IgM antibodies targeting neuronal surface proteins with the human natural killer epitope, which is contained in the myelin-associated glycoprotein, previously detected in serum of patients with neuropathy and monoclonal gammopathy, have been found in P-OMS patients.^[6]^ The role of the cell-mediated immune mechanism in the development of OMS is evidenced as well. The presence of the B-cells activating factor (BAFF) detected in serum and CSF and a higher CSF/serum BAFF ratio indicate intrathecal BAFF synthesis in the course of OMS.^[19,20]^ The response, both clinical and laboratory, to rituximab, which is a chimeric anti- B-lymphocyte antigen CD20 monoclonal antibody depleting circulating B cells, is confirmed in patients with OMS.^[5,21]^ Reported cases of OMS related to HIV infection prove T-cell contribution in the pathogenesis of the syndrome.^[22]^ In patients with OMS associated with recent HIV infections, the role of a decreased CD4/CD8 ratio is stressed. Apart from this, a rapid increase in CD4 cell counts may explain the appearance of OMS during HIV antiretroviral treatment.^[22–24]^

### 3.3 Clinical features of OMS

P-OMS, which accounts for more than 50% of all OMS cases, occurs in older people, aged 60. P-OMS is mainly associated with small cell lung carcinoma, breast adenocarcinoma and, in younger patients, ovarian teratoma.^[1–6,16,17]^ Less frequently, paraneoplastic OMS coexists with other types of tumors such as non-small cell lung carcinoma, sarcoma, chondrosarcoma, Hodgkin and non-Hodgkin lymphoma, carcinoma of the thyroid, thymus, uterus, and bladder, and melanoma.^[1–3,16]^ In more than 50% of cases, P-OMS precedes the diagnosis of a neoplasm, regardless of the type of tumor.^[6,18]^ According to the diagnostic criteria, P-OMS may be considered if the tumor is diagnosed within 5 years from the onset of paraneoplastic syndrome or if onconeuronal antibodies are present.^[25]^ Most patients with P-OMS do not have well characterized onconeuronal antibodies, except for women with breast adenocarcinoma and accompanying anti-Ri antibodies.^[5,6,18]^ Screening for a neoplasm with computer tomography and 18-fluoro-2-deoxyglucose-positron emission tomography of the chest, abdomen and pelvis, and regular follow-up are obligatory in cases with suspected P-OMS.^[1,10]^ A severe clinical course with encephalopathy, a disappointing response to immunotherapy, relapses, and poor prognosis enable a diagnosis of P-OMS.^[1,6,10,18]^

I-OMS more often affects younger people, aged 30 to 40, and may be preceded by infection or vaccination with flu-like symptoms.^[1,4,6,8,10,18]^ I-OMS of presumed para-infectious or postinfectious origin might be a form of brainstem encephalitis associated with viral (HIV, EBV, cytomegalovirus, varicella zoster virus, hepatitis C virus, Enterovirus, West Nile virus) or bacterial infections (Mycoplasma pneumonia, Salmonella enterica, Borrelia burgdorferi, Streptococcus).^[1,4,5,10,22,26]^ In young patients with OMS, the importance of the exclusion of HIV infection and ovarian teratoma is mentioned.^[6]^ OMS may occur in association with early HIV infection (seroconversion) or immune reconstitution syndrome in the course of antiretroviral therapy.^[6,22,27]^ Cases of OMS due to ovarian teratoma present with the clinical picture of P-OMS.^[6,28]^ A mild and monophasic clinical course with self-limited symptoms, a good response to immunotherapy and full recovery suggest I-OMS, although in older patients relapses and residual symptoms are sometimes observed.^[3,6,10]^

### 3.4 Treatment of OMS

Antineoplastic therapy alone (surgery, chemotherapy, and radiotherapy) is not effective in P-OMS. Antineoplastic therapy, together with immunotherapy, increases the chances of remission and improvement.^[1,3,4,16,18]^ In severe I-OMS, immunotherapy is the first line of treatment. Corticosteroids (prednisone, prednisolone, dexamethasone), adrenocorticotropic hormones, intravenous immunoglobulin, and plasma exchange could be used either alone or in combination; also, in combination with rituximab.^[3–6,8,18]^ In long-term treatment, immunosuppressants such as azathioprin, cyclophosphamide, and mofetil mycophenolate may be used.^[29,30]^ In para-infectious OMS, antimicrobiological treatment combined with immunotherapy is recommended.^[4]^ Additionally, as symptomatic treatment, regardless of OMS origin, antiepileptic drugs and benzodiazepines could be helpful. Antiepileptic drugs (levetiracetam, valproic acid, gabapentin, and topiramate) are indicated in patients with disabling myoclonus; benzodiazepines (clonazepam, nitrazepam) are crucial for opsoclonus treatment.^[1,4,10]^

### 3.5 OMS and pregnancy

In our case of a 30-year-old pregnant woman with severe OMS, we excluded a paraneoplastic, para-infectious etiology as well as metabolic, toxic, or structural impairment of CNS. The rapid progression of symptoms and changes in CSF were characteristic for immune-meditated mechanisms.^[17]^ The patient's relatively young age, her monophasic course, and response to immunotherapy with complete recovery may suggest an idiopathic origin of the disease.^[18]^ However, diagnosis of opsoclonus-myoclonus related to the pregnancy was highly likely. In the literature, we found only 3 other cases of OMS in the course of pregnancy in women in their 30s.^[31,32]^ The very rare cases of paraneoplastic OMS associated with ovarian and endometrial neoplasms and the 3 case reports of OMS during pregnancy may suggest the role of antigens within the Mullerian epithelium in female sexual organs as inciters of immune reactions responsible for OMS occurrence.^[16,31–33]^

In contrast to our patient with the onset of OMS in the 3rd month of pregnancy, previously reported cases developed neurological symptoms in the middle to late stages of pregnancy (5th, 7th, and 9th months).^[31,31]^ The clinical course of the disease with severe motor disability was similar in our and all cited cases. In electroencephalography, we registered a non-specific generalized slowdown of background activity, and bilateral paroxysmal theta waves, which might have been consistent with mild encephalopathy. In one of the previously described patients, CSF examination revealed lymphocytosis (9/mm^3^) and an elevated level of protein (60 mg/dl).^[31]^ We also observed a slightly elevated lymphocytosis (7/mm^3^) in CSF with a high IgG index, and oligoclonal IgG bands, which strongly suggested an intrathecal immunological reaction and immune-mediated movement disorder.

The pregnant women with OMS reported in the literature were treated with prednisolone, with positive effects. Improvement was observed during immunotherapy in the course of pregnancy, except for 1 case where treatment was introduced after delivery. Complete or nearly complete resolution of neurological symptoms was achieved in all cases with the end of pregnancy (childbirth or spontaneous miscarriage).^[31,32]^ In our OMS case, treatment with steroids and benzodiazepine was introduced during pregnancy, several days after onset of symptoms. After a week of therapy, the condition of the patient definitely improved. In the 6th month of pregnancy, the patient recovered completely and was completely independent. She remained asymptomatic in regular follow-ups. Achievement of improvement with reversibility of neurological symptoms could be associated with early initiation of immunotherapy and its synergistic effect with symptomatic treatment.^[3]^

OMS is a rare, disabling movement disorder mainly of an immune-mediated etiology. An understanding of the clinical symptoms and rare causes of OMS contributes to early diagnostic and therapeutic decisions, which ensures an optimal neurologic outcome. In the case of our patient, diagnosis of OMS related to the pregnancy could be made. Physiological change to immune system regulation during pregnancy might be a probable cause of OMS. However, the relationship between OMS and pregnancy remains uncertain and needs further investigations.

## Author contributions

**Conceptualization:** Magdalena Koszewicz, Ewa Koziorowska-Gawron.

**Data curation:** Ewa Koziorowska-Gawron, Joanna Bladowska.

**Formal analysis:** Maria Ejma.

**Funding acquisition:** Slawomir Budrewicz.

**Resources:** Maria Ejma.

**Visualization:** Joanna Bladowska.

**Writing – original draft:** Ewa Koziorowska-Gawron.

**Writing – review & editing:** Magdalena Koszewicz, Slawomir Budrewicz.
